# *N*,*N*′-Bis(4-chloro­benzyl­idene)-2,2-dimethyl­propane-1,3-diamine

**DOI:** 10.1107/S1600536808035307

**Published:** 2008-11-08

**Authors:** Hoong-Kun Fun, Hadi Kargar, Reza Kia

**Affiliations:** a X-ray Crystallography Unit, School of Physics, Universiti Sains Malaysia, 11800 USM, Penang, Malaysia; b Department of Chemistry, School of Science, Payame Noor University, Ardakan, Yazd, Iran

## Abstract

The title compound, C_19_H_20_Cl_2_N_2_, is a potential bidentate Schiff base ligand. Intra­molecular C—H⋯N hydrogen bonds form five-membered rings, generating *S*(5) ring motifs. Each imino functional group is coplanar with its adjacent benzene ring; the two benzene rings form a dihedral angle of 51.30 (4)°. An inter­esting feature of the crystal structure is weak inter­molecular Cl⋯Cl [3.4752 (4) Å] and Cl⋯N [3.2927 (9) Å] inter­actions. Inter­molecular Cl⋯N inter­actions link mol­ecules into dimers with *R_2_^2^(22)* ring motifs. The crystal structure is further stabilized by weak π–π [centroid–centroid distances = 3.6970 (6)–3.8560 (6) Å] inter­actions.

## Related literature

For hydrogen-bond motifs, see Bernstein *et al.* (1995 ▶). For related structures see, for example: Li *et al.* (2005 ▶); Bomfim *et al.* (2005 ▶); Glidewell *et al.* (2005 ▶, 2006 ▶); Sun *et al.* (2004 ▶); Fun *et al.* (2008 ▶).
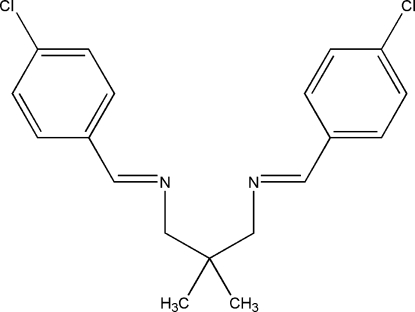


## Experimental

### 

#### Crystal data

C_19_H_20_Cl_2_N_2_
*M* *_r_* = 347.27
Monoclinic, 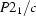

*a* = 19.6392 (3) Å
*b* = 9.3275 (2) Å
*c* = 9.7841 (2) Å
β = 92.213 (1)°
*V* = 1790.96 (6) Å^3^
*Z* = 4
Mo *K*α radiation
μ = 0.36 mm^−1^
*T* = 100 (1) K
0.51 × 0.35 × 0.10 mm

#### Data collection

Bruker SMART APEXII CCD area-detector diffractometer
Absorption correction: multi-scan (**SADABS**; Bruker, 2005 ▶) *T*_min_ = 0.836, *T*_max_ = 0.966
21670 measured reflections
6419 independent reflections
5273 reflections with *I* > 2σ(*I*)
*R*_int_ = 0.028

#### Refinement

*R*[*F* ^2^ > 2σ(*F* ^2^)] = 0.036
*wR*(*F* ^2^) = 0.095
*S* = 1.03
6419 reflections
208 parameters
H-atom parameters constrained
Δρ_max_ = 0.38 e Å^−3^
Δρ_min_ = −0.32 e Å^−3^

### 

Data collection: *APEX2* (Bruker, 2005 ▶); cell refinement: *APEX2* and *SAINT* (Bruker, 2005 ▶); data reduction: *SAINT*; program(s) used to solve structure: *SHELXTL* (Sheldrick, 2008 ▶); program(s) used to refine structure: *SHELXTL*; molecular graphics: *SHELXTL*; software used to prepare material for publication: *SHELXTL* and *PLATON* (Spek, 2003 ▶).

## Supplementary Material

Crystal structure: contains datablocks global, I. DOI: 10.1107/S1600536808035307/tk2319sup1.cif

Structure factors: contains datablocks I. DOI: 10.1107/S1600536808035307/tk2319Isup2.hkl

Additional supplementary materials:  crystallographic information; 3D view; checkCIF report

## Figures and Tables

**Table 1:** Hydrogen-bond geometry (Å, °)

*D*—H⋯*A*	*D*—H	H⋯*A*	*D*⋯*A*	*D*—H⋯*A*
C18—H18*B*⋯N1	0.96	2.60	2.9346 (15)	101
C19—H19*C*⋯N2	0.96	2.61	2.9416 (15)	101

**Fig. 1.**
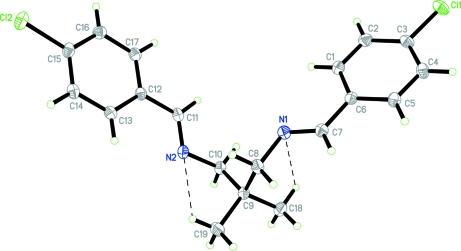
The molecular structure of (I) showing atom labels and 50% probability ellipsoids for non-H atoms. Intramolecular hydrogen bonds are shown as dashed lines.

## supplementary crystallographic information

### Comment

Schiff bases are one of most prevalent mixed-donor ligands in the field of coordination chemistry. They play an important role in the development of coordination chemistry related to catalysis and enzymatic reactions, magnetism, and supramolecular architectures. Structures of Schiff-base compounds derived from substituted benzaldehydes and closely related to the title compound, (I), have been reported previously (Li et al., 2005; Bomfim et al., 2005; Glidewell et al., 2005, 2006; Sun et al., 2004; Fun et al., 2008).

In (I), Fig. 1, each imino functional group is co-planar with its adjacent benzene ring. Intramolecular C—H···N  hydrogen bonds form five-membered rings, Fig. 1, producing S(5) ring motifs (Bernstein et al., 1995). The two benzene rings form a dihedral angle of 51.30 (4)°. The interesting feature of the crystal structure is the presence of weak intermolecular Cl···Cl [3.4852 (3) Å; symmetry code: x, 1 - y, -1/2 + z] and Cl···N [3.2927 (9) Å; symmetry code: -x, 1 - y, 1 - z] interactions. The intermolecular Cl···N interactions link neighbouring molecules into dimers with R_2_^2^(22) ring motifs (Bernstein et al., 1995). The crystal structure is further stabilized by weak intermolecular π–π interactions [Cg1···Cg1= 3.8560 (6) Å; symmetry code: 1 - x, 1 - y, 1 - z; Cg2···Cg2 = 3.6970 (6) Å; symmetry code: -x, 1 - y, 1 - z] (Cg1 and Cg2 are the centroids of the C1–C6 and C12–C17 rings, respectively.

### Experimental

The synthetic method has been described earlier (Fun *et al.*, 2008). Single crystals suitable for X-ray diffraction were obtained by evaporation of an ethanol solution of (I) held at room temperature.

### Refinement

All H atoms were included in the riding model approximation with C—H = 0.93 - 0.97 Å, and with *U*(H) = 1.2–1.5 times *U*_eq_(C).

### Crystal data

**Table:**

C_19_H_20_Cl_2_N_2_	*F*_000_ = 728
*M**_r_* = 347.27	*D*_x_ = 1.288 Mg m^−^^3^
Monoclinic, *P*2_1_/*c*	Mo *K*α radiation λ = 0.71073 Å
Hall symbol: -P 2ybc	Cell parameters from 8119 reflections
*a* = 19.6392 (3) Å	θ = 3.0–38.9º
*b* = 9.3275 (2) Å	µ = 0.36 mm^−^^1^
*c* = 9.7841 (2) Å	*T* = 100 (1) K
β = 92.213 (1)º	Plate, colourless
*V* = 1790.96 (6) Å^3^	0.51 × 0.35 × 0.10 mm
*Z* = 4	

### Data collection

**Table:**

Bruker SMART APEXII CCD area-detector diffractometer	6419 independent reflections
Radiation source: fine-focus sealed tube	5273 reflections with *I* > 2σ(*I*)
Monochromator: graphite	*R*_int_ = 0.028
*T* = 100.0(1) K	θ_max_ = 32.5º
φ and ω scans	θ_min_ = 1.0º
Absorption correction: multi-scan(*SADABS*; Bruker, 2005)	*h* = −29→29
*T*_min_ = 0.836, *T*_max_ = 0.966	*k* = −14→12
21670 measured reflections	*l* = −14→14

### Refinement

**Table:**

Refinement on *F*^2^	Secondary atom site location: difference Fourier map
Least-squares matrix: full	Hydrogen site location: inferred from neighbouring sites
*R*[*F*^2^ > 2σ(*F*^2^)] = 0.036	H-atom parameters constrained
*wR*(*F*^2^) = 0.095	*w* = 1/[σ^2^(*F*_o_^2^) + (0.0434*P*)^2^ + 0.5329*P*] where *P* = (*F*_o_^2^ + 2*F*_c_^2^)/3
*S* = 1.03	(Δ/σ)_max_ = 0.001
6419 reflections	Δρ_max_ = 0.38 e Å^−^^3^
208 parameters	Δρ_min_ = −0.32 e Å^−^^3^
Primary atom site location: structure-invariant direct methods	Extinction correction: none

### Special details

**Table:**

Experimental. The low-temperature data was collected with the Oxford Cyrosystem Cobra low-temperature attachment.
Geometry. All e.s.d.'s (except the e.s.d. in the dihedral angle between two l.s. planes) are estimated using the full covariance matrix. The cell e.s.d.'s are taken into account individually in the estimation of e.s.d.'s in distances, angles and torsion angles; correlations between e.s.d.'s in cell parameters are only used when they are defined by crystal symmetry. An approximate (isotropic) treatment of cell e.s.d.'s is used for estimating e.s.d.'s involving l.s. planes.
Refinement. Refinement of *F*^2^ against ALL reflections. The weighted *R*-factor *wR* and goodness of fit *S* are based on *F*^2^, conventional *R*-factors *R* are based on *F*, with *F* set to zero for negative *F*^2^. The threshold expression of *F*^2^ > σ(*F*^2^) is used only for calculating *R*-factors(gt) *etc*. and is not relevant to the choice of reflections for refinement. *R*-factors based on *F*^2^ are statistically about twice as large as those based on *F*, and *R*- factors based on ALL data will be even larger.

### Fractional atomic coordinates and isotropic or equivalent isotropic displacement parameters (Å^2^)

**Table:**

	*x*	*y*	*z*	*U*_iso_*/*U*_eq_	
Cl1	0.497643 (14)	0.49166 (3)	0.17717 (3)	0.02553 (7)	
Cl2	−0.166144 (13)	0.43828 (3)	0.54241 (3)	0.02640 (7)	
N1	0.28500 (4)	0.54904 (10)	0.70403 (9)	0.01950 (17)	
N2	0.14528 (4)	0.37002 (10)	0.85655 (9)	0.01864 (17)	
C1	0.35398 (5)	0.47808 (13)	0.45572 (11)	0.0216 (2)	
H1A	0.3155	0.4238	0.4731	0.026*	
C2	0.39180 (6)	0.44677 (13)	0.34258 (11)	0.0226 (2)	
H2A	0.3791	0.3720	0.2842	0.027*	
C3	0.44914 (5)	0.52935 (12)	0.31802 (11)	0.01962 (19)	
C4	0.46927 (5)	0.64046 (12)	0.40359 (11)	0.0208 (2)	
H4A	0.5080	0.6939	0.3863	0.025*	
C5	0.43069 (5)	0.67111 (12)	0.51594 (11)	0.01929 (19)	
H5A	0.4434	0.7466	0.5735	0.023*	
C6	0.37302 (5)	0.59009 (11)	0.54371 (10)	0.01710 (18)	
C7	0.33470 (5)	0.62450 (12)	0.66609 (11)	0.01815 (19)	
H7A	0.3474	0.7046	0.7176	0.022*	
C8	0.25179 (5)	0.59532 (12)	0.82751 (11)	0.0202 (2)	
H8A	0.2046	0.6187	0.8048	0.024*	
H8B	0.2740	0.6813	0.8628	0.024*	
C9	0.25474 (5)	0.47832 (12)	0.93875 (11)	0.01860 (19)	
C10	0.21738 (5)	0.34242 (12)	0.88813 (11)	0.01881 (19)	
H10A	0.2386	0.3070	0.8068	0.023*	
H10B	0.2216	0.2688	0.9579	0.023*	
C11	0.12219 (5)	0.33583 (11)	0.73837 (11)	0.01765 (18)	
H11A	0.1515	0.2936	0.6777	0.021*	
C12	0.05057 (5)	0.36042 (11)	0.69311 (10)	0.01656 (18)	
C13	0.00695 (5)	0.44318 (12)	0.77071 (11)	0.01855 (19)	
H13A	0.0230	0.4832	0.8529	0.022*	
C14	−0.06007 (5)	0.46587 (12)	0.72583 (11)	0.01985 (19)	
H14A	−0.0891	0.5207	0.7774	0.024*	
C15	−0.08314 (5)	0.40546 (12)	0.60260 (11)	0.01940 (19)	
C16	−0.04087 (5)	0.32318 (12)	0.52386 (11)	0.0204 (2)	
H16A	−0.0571	0.2832	0.4418	0.024*	
C17	0.02632 (5)	0.30141 (12)	0.56973 (11)	0.01898 (19)	
H17A	0.0553	0.2470	0.5175	0.023*	
C18	0.32900 (5)	0.43799 (14)	0.97383 (12)	0.0249 (2)	
H18A	0.3534	0.5214	1.0058	0.037*	
H18B	0.3497	0.4014	0.8937	0.037*	
H18C	0.3304	0.3660	1.0440	0.037*	
C19	0.22142 (6)	0.53832 (14)	1.06574 (12)	0.0261 (2)	
H19A	0.2452	0.6231	1.0960	0.039*	
H19B	0.2235	0.4678	1.1372	0.039*	
H19C	0.1747	0.5616	1.0435	0.039*	

### Atomic displacement parameters (Å^2^)

**Table:**

	*U*^11^	*U*^22^	*U*^33^	*U*^12^	*U*^13^	*U*^23^
Cl1	0.02940 (13)	0.02639 (15)	0.02128 (12)	0.00830 (10)	0.00692 (10)	0.00370 (10)
Cl2	0.01662 (11)	0.02663 (15)	0.03566 (15)	0.00011 (9)	−0.00260 (9)	0.00411 (11)
N1	0.0189 (4)	0.0204 (4)	0.0192 (4)	−0.0005 (3)	0.0014 (3)	0.0005 (3)
N2	0.0146 (3)	0.0200 (4)	0.0215 (4)	−0.0014 (3)	0.0025 (3)	0.0000 (3)
C1	0.0202 (4)	0.0231 (5)	0.0214 (5)	−0.0044 (4)	0.0001 (4)	−0.0006 (4)
C2	0.0251 (5)	0.0231 (5)	0.0197 (5)	−0.0028 (4)	−0.0007 (4)	−0.0026 (4)
C3	0.0221 (4)	0.0194 (5)	0.0175 (4)	0.0048 (4)	0.0024 (4)	0.0038 (4)
C4	0.0199 (4)	0.0176 (5)	0.0250 (5)	−0.0003 (4)	0.0039 (4)	0.0043 (4)
C5	0.0195 (4)	0.0154 (5)	0.0230 (5)	−0.0013 (3)	0.0012 (4)	0.0006 (4)
C6	0.0169 (4)	0.0167 (5)	0.0177 (4)	0.0001 (3)	−0.0004 (3)	0.0023 (4)
C7	0.0178 (4)	0.0174 (5)	0.0191 (4)	0.0002 (3)	−0.0002 (3)	0.0007 (4)
C8	0.0185 (4)	0.0197 (5)	0.0226 (5)	−0.0003 (4)	0.0044 (4)	−0.0008 (4)
C9	0.0162 (4)	0.0215 (5)	0.0182 (4)	−0.0019 (3)	0.0019 (3)	−0.0015 (4)
C10	0.0158 (4)	0.0191 (5)	0.0216 (5)	0.0002 (3)	0.0018 (3)	0.0000 (4)
C11	0.0165 (4)	0.0160 (5)	0.0207 (5)	0.0005 (3)	0.0049 (3)	0.0005 (4)
C12	0.0161 (4)	0.0153 (4)	0.0184 (4)	−0.0005 (3)	0.0028 (3)	0.0019 (3)
C13	0.0190 (4)	0.0185 (5)	0.0183 (5)	0.0007 (3)	0.0033 (3)	−0.0006 (4)
C14	0.0180 (4)	0.0189 (5)	0.0230 (5)	0.0017 (4)	0.0046 (4)	0.0015 (4)
C15	0.0156 (4)	0.0177 (5)	0.0249 (5)	−0.0010 (3)	0.0005 (3)	0.0049 (4)
C16	0.0215 (4)	0.0195 (5)	0.0202 (5)	−0.0020 (4)	−0.0003 (4)	−0.0008 (4)
C17	0.0194 (4)	0.0180 (5)	0.0197 (5)	0.0007 (3)	0.0029 (3)	−0.0006 (4)
C18	0.0186 (4)	0.0303 (6)	0.0255 (5)	−0.0024 (4)	−0.0025 (4)	0.0008 (4)
C19	0.0272 (5)	0.0292 (6)	0.0222 (5)	−0.0042 (4)	0.0065 (4)	−0.0055 (4)

### Geometric parameters (Å, °)

**Table:**

Cl1—C3	1.7411 (11)	C9—C19	1.5323 (15)
Cl2—C15	1.7390 (10)	C9—C10	1.5370 (15)
N1—C7	1.2703 (13)	C10—H10A	0.9700
N1—C8	1.4602 (14)	C10—H10B	0.9700
N2—C11	1.2664 (14)	C11—C12	1.4761 (14)
N2—C10	1.4603 (13)	C11—H11A	0.9300
C1—C2	1.3876 (15)	C12—C17	1.3939 (15)
C1—C6	1.3956 (15)	C12—C13	1.3987 (14)
C1—H1A	0.9300	C13—C14	1.3876 (14)
C2—C3	1.3929 (15)	C13—H13A	0.9300
C2—H2A	0.9300	C14—C15	1.3908 (16)
C3—C4	1.3805 (16)	C14—H14A	0.9300
C4—C5	1.3886 (14)	C15—C16	1.3860 (15)
C4—H4A	0.9300	C16—C17	1.3921 (14)
C5—C6	1.3970 (14)	C16—H16A	0.9300
C5—H5A	0.9300	C17—H17A	0.9300
C6—C7	1.4741 (14)	C18—H18A	0.9600
C7—H7A	0.9300	C18—H18B	0.9600
C8—C9	1.5408 (16)	C18—H18C	0.9600
C8—H8A	0.9700	C19—H19A	0.9600
C8—H8B	0.9700	C19—H19B	0.9600
C9—C18	1.5322 (15)	C19—H19C	0.9600
			
C7—N1—C8	116.79 (9)	C9—C10—H10A	109.3
C11—N2—C10	117.31 (9)	N2—C10—H10B	109.3
C2—C1—C6	120.72 (10)	C9—C10—H10B	109.3
C2—C1—H1A	119.6	H10A—C10—H10B	107.9
C6—C1—H1A	119.6	N2—C11—C12	122.56 (9)
C1—C2—C3	118.84 (10)	N2—C11—H11A	118.7
C1—C2—H2A	120.6	C12—C11—H11A	118.7
C3—C2—H2A	120.6	C17—C12—C13	119.48 (9)
C4—C3—C2	121.67 (10)	C17—C12—C11	119.22 (9)
C4—C3—Cl1	118.62 (8)	C13—C12—C11	121.30 (9)
C2—C3—Cl1	119.71 (9)	C14—C13—C12	120.38 (10)
C3—C4—C5	118.84 (10)	C14—C13—H13A	119.8
C3—C4—H4A	120.6	C12—C13—H13A	119.8
C5—C4—H4A	120.6	C13—C14—C15	119.07 (10)
C4—C5—C6	120.92 (10)	C13—C14—H14A	120.5
C4—C5—H5A	119.5	C15—C14—H14A	120.5
C6—C5—H5A	119.5	C16—C15—C14	121.61 (9)
C1—C6—C5	119.00 (9)	C16—C15—Cl2	118.88 (9)
C1—C6—C7	122.08 (9)	C14—C15—Cl2	119.47 (8)
C5—C6—C7	118.91 (9)	C15—C16—C17	118.83 (10)
N1—C7—C6	122.61 (10)	C15—C16—H16A	120.6
N1—C7—H7A	118.7	C17—C16—H16A	120.6
C6—C7—H7A	118.7	C16—C17—C12	120.63 (9)
N1—C8—C9	111.67 (9)	C16—C17—H17A	119.7
N1—C8—H8A	109.3	C12—C17—H17A	119.7
C9—C8—H8A	109.3	C9—C18—H18A	109.5
N1—C8—H8B	109.3	C9—C18—H18B	109.5
C9—C8—H8B	109.3	H18A—C18—H18B	109.5
H8A—C8—H8B	107.9	C9—C18—H18C	109.5
C18—C9—C19	109.91 (9)	H18A—C18—H18C	109.5
C18—C9—C10	107.95 (9)	H18B—C18—H18C	109.5
C19—C9—C10	110.43 (8)	C9—C19—H19A	109.5
C18—C9—C8	109.97 (8)	C9—C19—H19B	109.5
C19—C9—C8	107.97 (9)	H19A—C19—H19B	109.5
C10—C9—C8	110.61 (9)	C9—C19—H19C	109.5
N2—C10—C9	111.70 (9)	H19A—C19—H19C	109.5
N2—C10—H10A	109.3	H19B—C19—H19C	109.5
			
C6—C1—C2—C3	−0.13 (17)	C11—N2—C10—C9	125.72 (10)
C1—C2—C3—C4	0.35 (17)	C18—C9—C10—N2	178.07 (9)
C1—C2—C3—Cl1	179.70 (9)	C19—C9—C10—N2	57.90 (12)
C2—C3—C4—C5	−0.74 (17)	C8—C9—C10—N2	−61.57 (11)
Cl1—C3—C4—C5	179.89 (8)	C10—N2—C11—C12	−178.94 (9)
C3—C4—C5—C6	0.93 (16)	N2—C11—C12—C17	−170.66 (10)
C2—C1—C6—C5	0.31 (17)	N2—C11—C12—C13	10.16 (16)
C2—C1—C6—C7	−178.88 (10)	C17—C12—C13—C14	0.35 (16)
C4—C5—C6—C1	−0.72 (16)	C11—C12—C13—C14	179.52 (10)
C4—C5—C6—C7	178.50 (10)	C12—C13—C14—C15	−0.14 (16)
C8—N1—C7—C6	179.80 (9)	C13—C14—C15—C16	0.08 (16)
C1—C6—C7—N1	4.79 (16)	C13—C14—C15—Cl2	−177.78 (8)
C5—C6—C7—N1	−174.40 (10)	C14—C15—C16—C17	−0.23 (16)
C7—N1—C8—C9	−120.74 (10)	Cl2—C15—C16—C17	177.64 (8)
N1—C8—C9—C18	57.83 (12)	C15—C16—C17—C12	0.44 (16)
N1—C8—C9—C19	177.75 (8)	C13—C12—C17—C16	−0.51 (16)
N1—C8—C9—C10	−61.31 (11)	C11—C12—C17—C16	−179.70 (10)

### Hydrogen-bond geometry (Å, °)

**Table:**

*D*—H···*A*	*D*—H	H···*A*	*D*···*A*	*D*—H···*A*
C18—H18B···N1	0.96	2.60	2.9346 (15)	101
C19—H19C···N2	0.96	2.61	2.9416 (15)	101
